# Anomalous brain gyrification patterns in major psychiatric disorders: a systematic review and transdiagnostic integration

**DOI:** 10.1038/s41398-021-01297-8

**Published:** 2021-03-17

**Authors:** Daiki Sasabayashi, Tsutomu Takahashi, Yoichiro Takayanagi, Michio Suzuki

**Affiliations:** 1 Department of Neuropsychiatry, University of Toyama Graduate School of Medicine and Pharmaceutical Sciences, Toyama, Japan; 2 Research Center for Idling Brain Science, University of Toyama, Toyama, Japan; 3 Arisawabashi Hospital, Toyama, Japan

**Keywords:** Neuroscience, Predictive markers, Psychiatric disorders, Diagnostic markers

## Abstract

Anomalous patterns of brain gyrification have been reported in major psychiatric disorders, presumably reflecting their neurodevelopmental pathology. However, previous reports presented conflicting results of patients having hyper-, hypo-, or normal gyrification patterns and lacking in transdiagnostic consideration. In this article, we systematically review previous magnetic resonance imaging studies of brain gyrification in schizophrenia, bipolar disorder, major depressive disorder, and autism spectrum disorder at varying illness stages, highlighting the gyral pattern trajectory for each disorder. Patients with each psychiatric disorder may exhibit deviated primary gyri formation under neurodevelopmental genetic control in their fetal life and infancy, and then exhibit higher-order gyral changes due to mechanical stress from active brain changes (e.g., progressive reduction of gray matter volume and white matter integrity) thereafter, representing diversely altered pattern trajectories from those of healthy controls. Based on the patterns of local connectivity and changes in neurodevelopmental gene expression in major psychiatric disorders, we propose an overarching model that spans the diagnoses to explain how deviated gyral pattern trajectories map onto clinical manifestations (e.g., psychosis, mood dysregulation, and cognitive impairments), focusing on the common and distinct gyral pattern changes across the disorders in addition to their correlations with specific clinical features. This comprehensive understanding of the role of brain gyrification pattern on the pathophysiology may help to optimize the prediction and diagnosis of psychiatric disorders using objective biomarkers, as well as provide a novel nosology informed by neural circuits beyond the current descriptive diagnostics.

## Introduction

Previous studies demonstrated that established psychiatric disorders, such as schizophrenia (SCZ), bipolar disorder (BD), major depressive disorder (MDD), and autism spectrum disorder (ASD), are generally associated with neurodevelopmental pathology^1–4^, leading the debate as to whether they signify distinct entities that possess overlapping foundations or are different variants of one underlying disease. These major psychiatric disorders are presently differentiated based on their specific clinical representations, but a part of their symptoms transcends the diagnostic categories. A transdiagnostic approach may be useful for accurate diagnosis in the initial stage of illnesses and for clarifying the nature of their relationships among disorders (especially common and distinct mechanisms under their respective semiotics)^5^. Although preceding studies revealed shared genetic variations within a range of psychiatric disorders^6^, including common genetic-risk loci between SCZ and BD reported in a recent large-scale genome-wide association study (GWAS)^7^, it remains unclear how the disorders are trans-diagnostically characterized by neuroanatomical features, i.e., inter alia gyrification patterns. Thus, the common and distinct alterations in gyrification pattern across major psychiatric disorders have yet to be established.

Anomalous patterns of brain gyrification are assumed to be a consequence of pre/perinatal neurodevelopmental insult because cortical folding starts at 10 weeks of gestation to the third trimester of pregnancy (primary gyri emerge until 28 weeks of gestation and higher-order gyri develop thereafter) and its pattern remains rather stable after birth^8,9^. Very preterm children and adults showed significant changes in brain gyrification pattern^10,11^ that were associated with cognitive and psychopathology scores^11^, suggesting the impact of obstetric complications for the development of brain gyrification. As obstetric complications such as perinatal hypoxia and preterm birth were associated with locally reduced gyrification in psychosis^12,13^, the abnormal microenvironment in utero may impact the development of gyrification deficits which might be related to future psychopathology. Contrary to gray matter volume, which can be affected by age^14^, disease process^15^, and psychotropic drugs^16^, cortical gyrification patterns may be a more static neurodevelopmental marker. Therefore, it may be useful for feasible early intervention^17^. Although the gyral patterns may be a static neurodevelopmental marker, recent studies propounded a putative gyral pattern trajectory model that healthy individuals have a non-linear decline of gyrification index (GI) with age^18,19^. Patients with schizophrenia may also exhibit GI reduction over time^20^, suggesting multiple pathological processes at varying clinical stages. Although it is largely unknown whether patients with other psychiatric disorders exhibit gyral pattern changes during the course of the illness, such a potential longitudinal change may partly explain the conflicting results of patients with psychiatric disorders having hyper-, hypo-, or normal gyrification patterns^18,19,21–36^.

We systematically reviewed recent neuroimaging findings of anomalous gyral patterns in major psychiatric disorders at varying illness stages. This review article aims to integrally understand the gyral findings by focusing on the gyral pattern trajectories. Moreover, we propose an overarching model that spans the diagnoses through the transdiagnostic considerations, which may aid in clinical diagnosis and prediction of the disorders, and also transcend the limitations of the established nosological systems.

## Magnetic resonance imaging (MRI) findings of brain gyrification

### Study selection

We aimed to systematically review the studies published between May 1, 1997 and June 30, 2019 that investigated the gyral pattern in major psychiatric disorders, including the high-risk state both clinically and genetically for psychosis, by following the Preferred Reporting Items for Systematic Reviews and Meta-Analyses (PRISMA) statement^37^. We searched for studies in PubMed and Google Scholar using the terms (schizophrenia OR bipolar disorder OR major depressive disorder OR autism spectrum disorder OR high risk) AND (gyrification OR local gyrification index). We further performed a cited reference search starting with the identified studies in Web of Science, and added several relevant reports. The searches were rerun just before the final analyses in June 2020. Two of the authors (D.S. and Y.T.) independently conducted a hand search and resolved their disagreement in study selection by consensus.

**Fig. 1:**
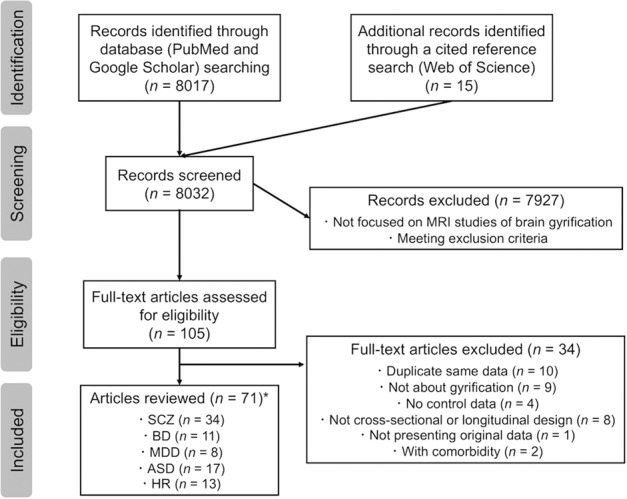
Preferred reporting items for systematic reviews and meta-analyses (PRISMA) diagram for study search. *Ten studies investigated the gyrification patterns in two or three psychiatric conditions (major psychiatric disorder or high-risk state for psychosis). ASD autism spectrum disorder, BD bipolar disorder, HR high risk for psychosis, MDD major depressive disorder; MRI magnetic resonance imaging, SCZ schizophrenia.

Only full-length or short articles written in English were selected. We included case-control studies (cross-sectional or longitudinal designs) with MRI data in patients with major psychiatric disorders, individuals at high-risk for psychosis, and healthy controls. Patients met the Diagnostic and Statistical Manual of Mental Disorders (DSM), 3rd, 4th, or 5th edition criteria or the International Classification of Disease diagnostic (ICD), 10th revision criteria for each psychiatric disorder. In addition, high-risk individuals were siblings of the patients or met the diagnosis criteria, such as the Comprehensive Assessment of At-Risk Mental States (CAARMS)^38^ and the Structured Interview for Prodromal Symptoms /the Scale of Prodromal Symptoms (SIPS/SOPS)^39^. All studies that investigated psychiatric or physical conditions other than the aforementioned states were excluded. Studies using non-T1-weighted protocols were also excluded. In all, we included 71 articles (Fig. 1), which formed the empirical basis of this review.

The quality assessment of the literature was conducted by using the Newcastle-Ottawa Quality Assessment Scale (NOS)^40^ arranged for a case-control study design. This scale contains six items, categorized into two dimensions including selection and comparability, ranging between zero (the lowest quality) up to six (the highest quality) stars. In this review, the NOS stars ranged from four to six and the average was 5.6 (Supplemental Table S1), suggesting that the quality of the included studies was relatively high on average.

### Development of measures to assess cortical folding

In earlier studies, the GI, the ratio of the total folded cortical surface over the perimeter of the brain that was manually traced on 2-D coronal sections^41^, was standardly used to quantify cortical folding due to its easy interpretation and implemental simplicity. After that, the automated GI (A-GI) was developed by adapting the GI to a fully automated 2-D method in order to improve reliability and reproducibility in larger cohorts^42^. However, these methods were two-dimensional analyses for limited brain areas, which may be biased by slice orientation, buried sulci, and anomalies of sublobe regions. With the recent technological developments in 3-D image reconstruction and surface-based morphometry, the measurement of local GI (LGI) has widely been accepted in psychiatric researches. The index is calculated by dividing the pial surface area by the corresponding outer surface area for each vertex^43^. The measurement of LGI has methodological advantages over other methods by taking into account the inherent three-dimensional nature of the entire cortical surface. Several studies employed other approaches, such as the mean curvature computed by normalized summation of the amplitude and frequency of the simulated folding^44^ and the sulcal index (SI), which is calculated by the ratio between the sulcal area and the area of the corresponding outer cortex^45^, while using the same 3-D mesh of the cortical surface.

### Lifetime trajectory of brain gyrification pattern in healthy subjects

MRI studies in healthy subjects, including longitudinal assessments, revealed the typical developmental trajectory of the GI. After birth, the entire cortical GI increased and reached its peak by 2 years of age (approximately 20% increase at 2 years)^46^, and then decreased with decreasing velocity thereafter^18,47^. During adolescence, the GI was reported to decline steadily with an annual decrease of around 1%, especially in the frontal and occipital cortices, where sulcal widening and reduced sulcal depth occurred together^48^.

### Findings in individuals at high risk for psychosis

Several MRI studies have reported anomalous patterns of brain gyrification prior to the onset of overt psychotic symptoms (Supplemental Table S2). An increased GI in the prefrontal cortex, especially on the right hemisphere, was reported in siblings of SCZ patients, especially in those who subsequently developed SCZ, compared with healthy controls^49–51^. Individuals with at-risk mental state (ARMS) have an increased GI in widespread cortical areas regardless of the outcome (i.e., future transition or non-transition to psychosis)^17,24^, whereas the left occipital hyper-gyrification pattern may be specific to the ARMS subgroup who later develop psychosis^17^. Subjects with schizotypal features, who are also at increased risk for developing psychosis, exhibit an increased GI in the prefrontal and parietal cortices^52,53^.

### Findings in patients with major psychiatric disorders

**Fig. 2:**
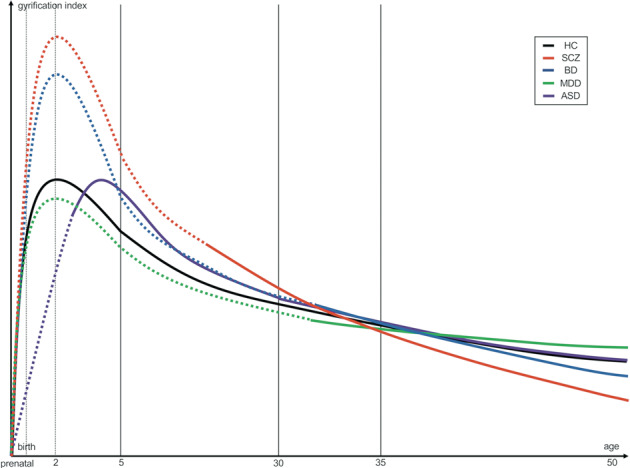
Putative gyral pattern trajectories of the frontal cortex in major psychiatric disorders. Dotted line: Presumed trajectory of gyrification pattern suggested from findings reported in neighboring developmental periods and other indicators (e.g., gene expression, gray matter volume, and white mater integrity); Solid line: Dominant trajectory of gyrification pattern based on previous gyral findings being weighted according to the number of studies, sample size for studies, representativeness of cases, and methodological advantage to assess gyral patterns. ASD autism spectrum disorder, BD bipolar disorder, HC healthy controls, MDD major depressive disorder, SCZ schizophrenia.

Based on previous MRI findings being weighted according to the number of studies, sample size for studies, representativeness of cases (one of the NOS components), and methodological advantage to assess gyral patterns [priority to the measures using 3-D mesh model of the cortical surface (e.g., LGI, mean curvature, SI)], the gyrification pattern trajectories in each illness were summarized in Fig. 2 and Supplemental Tables S3-6. We combined the results of region-of-interest (ROI) -based studies, which could have overlooked anomalous gyral patterns outside the ROI, and whole-brain voxel- or vertex- wised studies, but the summary findings remained essentially the same even when we excluded 8 ROI-based studies and assessed only 53 whole-brain analyses.

Adolescent and young adult patients with SCZ exhibit an increased GI or curvature in the frontal and other widespread regions compared with healthy controls^23,24,28,32,36,51,54–58^, whereas patients in their mid-thirties and older mostly exhibit a decreased GI or SI in widespread cortical areas, particularly on the frontal cortices^18,26,45,59–64^. Although few MRI studies have examined the anomalous gyral patterns in mood disorders during adolescence and young adulthood, patients with BD likely exhibit increased frontal curvature during their mid-thirties compared with healthy controls^28^, but have reduced frontal GI or SI in their early forties and afterwards^21,61^. Compared with healthy controls, patients with MDD exhibit reduced frontal GI during their thirties^22,33^, and then increased frontal GI around their forties and afterwards^27,31,65^. Patients with ASD have delayed gyral development compared with typically developing controls during infancy^19^, transition to an increased GI in widespread cortical regions during childhood^25,30,35,66–68^, and lastly approach normal gyrification pattern in adulthood and thereafter^4,29,34^. Given the lack of longitudinal evidence, this framework is not able to explain why different trajectories should occur in disorders with differing phenotypes, and our model (Fig. 2) is amenable for further revisions.

In addition, we divided the patients with major psychiatric disorders (except the ASD) into those with early (illness duration <3 years), middle (3–10 years), and late (10 years and afterwards) illness stages, and then conducted a subgroup analysis on the basis of 33 relevant MRI studies to see how the disease process could impact on gyrification pattern changes (Supplemental Fig. S1); the results supported the role of illness stages on gyrification pattern for these psychiatric disorders.

### Relationship of brain gyrification pattern with clinical symptoms and cognitive performance (Supplemental Table S7)

The severity of positive psychotic symptoms, including verbal hallucination, in SCZ was reported to be correlated with LGI in the right frontal and temporo-limbic region^32,57^. SCZ patients with persistent negative symptoms (i.e., deficit subtype) are characterized by greater frontal and lesser parietal hyper-gyrification patterns compared with those without deficit syndrome^36^. Neurological soft signs (NSS) in SCZ patients, which are subtle neurological deficits in the execution of motor or sensory tasks^69^ and potentially link to cerebellar gyral changes^70^, are negatively associated with LGI in widespread cortical areas^29^. In patients with MDD, the severity of depressive mood and number of depressive episodes are inversely correlated with LGI in the lateral frontal cortices, lateral parietal cortices, and fusiform gyri^33,65^. Autistic symptoms, especially communication impairments and repetitive behaviors, are significantly correlated with the right parietal GI in patients with ASD and in their unaffected co-twins^19,67,71^. The right prefrontal GI is correlated not only with general cognition in patients with different mental states^52,61,67^, but also with specific cognitive subdomains such as executive function in SCZ^32^, working memory in BD^61^, and metacognition in ASD^35^.

## Mechanism of brain gyrification

Developmental mechanisms of cortical folding, which are necessary for obtaining higher brain functions^72^, remain elusive. However, rapid and efficient genetic manipulation techniques in animals, in addition to computational neuroscience approaches, have been employed to clarify the mechanisms^73^. Moreover, neuroimaging studies in patients with neuropsychiatric disorders have reported associations of gyral pattern changes with various genotypes^31,74,75^. Here, we propose possible mechanisms of brain gyrification by dividing the gyral constructions into (1) primary gyri and (2) higher-order gyri. We further discuss how the upstream factors controlling cortical folding are involved in forming disease-specific gyral pattern trajectories.

### Primary gyri—molecular-level model

**Fig. 3:**
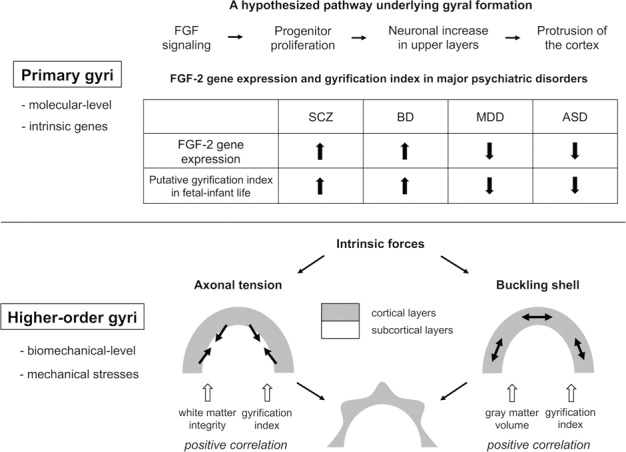
Assumed mechanisms of brain gyrification. Upper: Molecular-level mechanisms in primary gyri (mainly affected by intrinsic genes). Below: Biomechanical-level mechanisms in higher-order gyri (mainly affected by mechanical stress). ASD autism spectrum disorder, BD bipolar disorder, FGF fibroblast growth factor, MDD major depressive disorder, SCZ schizophrenia.

As the positions of primary gyri are roughly the same among individuals^76^, their folding patterns may be mainly determined by intrinsic genetic mechanisms. Although previous studies have shown that main gyral formation can be modulated by several genes such as *Cdk5*^77^ and *Tbr2*^78^, we focus on the fibroblast growth factor (FGF) gene in the present review, because of a relative abundance of literature that reported its relevance to psychiatric disorders. However, the genetic mechanism of FGF-2 gene should be considered with caution, because this gene was not hit by GWAS^7^. Altered expression of FGF-2 gene could be a consequence of other upstream processes and it would also have more downstream consequences. FGF-2 and its coupled FGF receptors (FGFRs), whose genes were differentially expressed throughout the central nervous system tissues, were reported to regulate neuronal proliferation and differentiation (on neuronal precursor cells) as well as axonal sprouting and ensheathment (on oligodendrocytes) during a developmental stage^79,80^. Later in life, FGF-2 and FGFRs are thought to be involved in the maintenance of neuronal tissue (on tissue lesions) as well as learning and memory (on hippocampal pyramidal cells)^79^. Mice lacking FGF-2, FGFR-1, and FGFR-2 expressions showed psychosis-related behavior, cognitive deficits, and increased anxiety that coincided with cortical volume reduction, while administration of FGF-2 could ameliorate depression-like behaviors in animals^81^. A series of experimental approaches using gyrencephalic mammal ferrets^82–85^ led to a hypothesized pathway, where FGF signaling induces progenitor cell proliferation in the subventricular zone (SVZ), resulting in a neuronal increase preferentially in cortical upper layers that is responsible for protrusion of the cortex (Fig. 3).

While multiple genes regulate SVZ progenitors in the developing cerebral cortex^86^, overexpression of the FGF-2 gene in the dorsolateral prefrontal cortex^87^ commonly observed in patients with SCZ and BD may be implicated in increased GI during their cortical development. Some findings of elevated FGF-2 serum protein level in both SCZ and BD^88–90^, which could be directly related to FGF-2 level in brain capillaries through blood-brain barrier^91^, might also reinforce the evidence suggestive of an increased FGF-2 signaling in brain. In contrast, reduced FGF-2 transcripts in the frontal cortex and hippocampus in patients with MDD^92,93^, and lower level of serum FGF-2 in children with ASD^94^ may be related to reduced GI during gyral formation.

### Higher-order gyri—biomechanical-level model

The positions of higher-order gyri are less uniformly distributed among individuals, suggesting that they are affected by mechanical stress rather than genetic loads. Two mechanistic explanations focusing on the intrinsic forces may be mainly involved in gyral pattern changes after childhood (Fig. 3). One is a “buckling shell” model: if the surface shell (e.g., cortical layers) expands more rapidly than the core shell (e.g., subcortical layers), then the shell eventually buckles^95^. Another is an “axonal tension” model: tension along obliquely oriented axonal trajectories between adjacent cortical regions may cause tangential force components that induce the cortical folds^96^. These potential mechanisms may also be modulated by a number of genes implicated in several developmental processes of the brain^97^.

As gray matter volume^98^ and white matter integrity^71,99^ are positively correlated with GI under both healthy and pathological conditions, the gyrification pattern trajectory unique to each disorder may be partly explained by longitudinal changes in cortical morphology and/or white matter pathology.

Patients with SCZ during adolescence and adulthood likely exhibit progressive gray matter reduction in the fronto-temporo-parietal region^100–102^ in addition to white matter integrity disruption in the peri-sylvian area^103^. BD patients also exhibit prefrontal gray matter volume loss over time^104^, although the degree of loss is smaller than that in SCZ^105^. These findings may reflect the longitudinal GI decrease in the corresponding regions. Faster global cortical enlargement during childhood^106^ and slower frontotemporal gray matter expansion thereafter^107^ in ASD patients than in typically developing controls may affect the gyral pattern trajectory of ASD. Patients with MDD during late adulthood demonstrate neither gray matter reduction over time^108^ nor white matter microstructural alterations^109^, supporting the low reduction rate of GI around the frontal region.

## Implication of anomalous gyral pattern

### Gyrification pattern and neural connectivity

An experimental study on the consequences of prefrontal resection revealed the eruption of additional fissures around the lesion and rerouting of intact cortico-striatal projections^110^. Recent multimodal approaches demonstrated that LGI alterations and changes in the degree centrality (DC)^64^ or radial diffusivity^99^ were located in the overlapping region in SCZ patients, and further that speech connectedness correlating with LGI and DC explained a thought disturbance, cognitive impairment, and functional outcome, irrespective of diagnostic boundaries^111^. Taken together, it is suggested that abnormalities in local GI are related to brain dysconnectivity of the corresponding area as neural substrates for various clinical symptoms and cognitive performance.

### Brain dysconnectivity in major psychiatric disorders

Previous neuroimaging studies demonstrated disrupted functional or structural connectivity in numerous psychiatric disorders by adopting a transdiagnostic approach^5^.

Both SCZ and BD patients have compromised connectivity centered on the frontal region^112,113^, but the hypo-connectivity within the frontoparietal executive network may be more marked in SCZ patients than in BD patients^114^. Young patients with SCZ and ASD likely exhibit shared atypical default mode network (DMN) and salience network (SN) functional connectivities, which are related to the social functioning score^115^. A large-scale multidiagnostic comparison of diffusion tensor imaging (DTI) data suggested white matter microstructural abnormalities of the corpus callosum commonly in SCZ, BD, and ASD patients, possibly reflecting disturbed extensive networks subserving emotional regulation^116^, but alterations of the tracts connecting neocortical areas may be specific to SCZ patients^109^. On the other hand, MDD patients do not have altered white matter connectivity^109^. Functional disconnectivity in the frontal and temporo-limbic regions may underlie positive psychotic symptoms in SCZ^117,118^, whereas altered intracortical couplings in frontoparietal brain circuits may be associated with prominent negative symptoms^119^. Children with ASD were reported to have increased short-range connectivities within the frontal lobe^120^ but reduced long-range connectivities across different brain areas^121^. Among them, altered functional connectivities of the somatosensory cortex, which are considered to be disease-specific changes, may be associated with autistic symptoms such as social communication deficits^122^.

In summary, patients with major psychiatric disorders may have both common and distinct connectivity abnormalities in neural circuits involving several brain regions, some of which can explain certain clinical features.

### Overarching model that spans the diagnoses

**Fig. 4:**
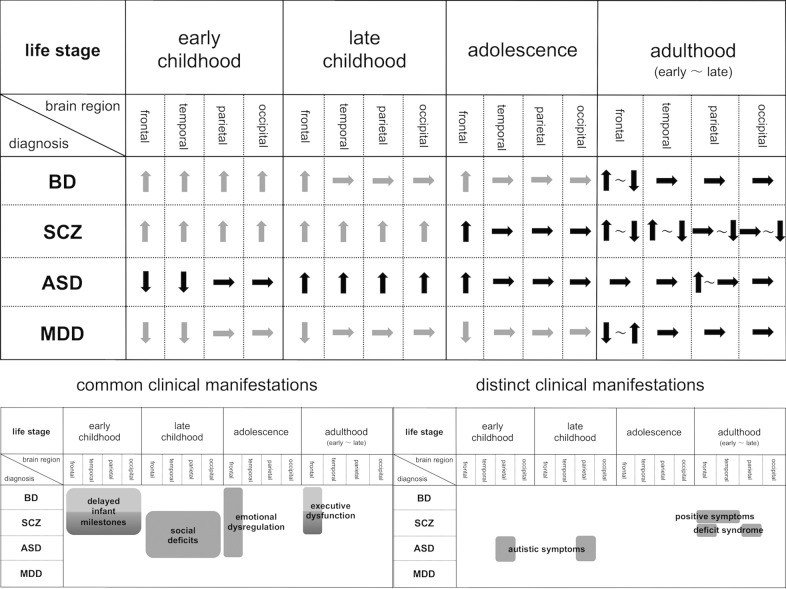
Overarching model that spans the diagnoses. Upper: The gyrification findings in patients with major psychiatric disorders at varying stages of life compared with healthy controls. Gray arrow: Presumed direction of gyral pattern changes suggested from findings reported in neighboring developmental periods and other indicators (e.g., gene expression, gray matter volume, and white mater integrity); Black arrow: Dominant direction of gyral pattern changes based on previous gyral findings being weighted according to the number of studies, sample size for studies, representativeness of cases, and methodological advantage to assess gyral patterns. Below: Concept maps showing common or distinct clinical manifestations mapped onto deviated gyrification patterns across psychiatric disorders. ASD autism spectrum disorder, BD bipolar disorder, MDD major depressive disorder, SCZ schizophrenia.

Based on the patterns of local connectivity and variations in neurodevelopmental gene expression across disorders described above, commonly and differentially altered gyrification patterns in patients with major psychiatric disorders may represent common and distinct clinical manifestations. We propose an overarching model that spans the diagnoses to clarify how deviated gyral pattern trajectories map onto clinical manifestations (Fig. 4).

### Common clinical manifestations

#### SCZ and BD

As the NSS in SCZ patients is correlated with both LGI^29^ and delayed childhood development^123^, the widespread hyper-gyrification pattern in early childhood presumed in SCZ and BD patients may underlie their delayed infant milestones (e.g., not walking or speaking at year 1)^124^. Such a delay is greater in SCZ patients than in BD patients^125^, probably due to the greater genetic burden of neurodevelopmental risk in SCZ patients^126^. Longitudinal reductions of the frontal GI around early adulthood and thereafter commonly observed in SCZ and BD patients (more prominent changes in SCZ patients than in BD patients) may be associated with progressive frontal gray matter reduction that is also commonly observed in SCZ^102^ and BD^104^, and is related to executive dysfunction^127^. As transdiagnostic impairments mainly in the anterior-cingulo-insular network in chronic SCZ and BD patients link to general executive dysfunction^128^, the cognitive deficit may result from common frontal-related disconnection.

#### SCZ and ASD

The shared widespread hyper-gyrification pattern suggested in SCZ and ASD patients during late childhood is expected to be related to social deficits (e.g., impaired social interaction, disabilities in communication, and restricted interests). The oxytocin system is known to play a key role in social cognition and behavior^129^. The oxytocin receptor (OXTR) genotype was reported to affect brain functional connectivity^130^. As a neural correlate of social deficits shared in ASD and SCZ^114^, disturbed functional connectivities in diverse cortical regions, including DMN/SN-related brain regions, may partly be derived from genetic variations in the OXTR gene^130^, which are associated with the risk of both disorders^131,132^.

#### SCZ, BD, and ASD

Emotional dysregulation during adolescence, which is commonly observed in conventionally defined diagnoses, may be explained in part by the hyper-gyrification pattern common to SCZ, BD, and ASD patients in the frontal region. Such dysregulation may be mediated by abnormal emotional regulation circuitry (e.g., uncinate fasciculus and forceps minor)^133^. A common relationship between right prefrontal GI and general cognition in a range of psychiatric disorders suggests a neural mechanism shared across diseases^134^, whereas distinct relationships between right prefrontal GI and cognitive subdomains by each disorder can reflect a set of discrete dysfunctions^135^. Thus, further studies are required to examine which hypotheses explain the associations between anomalous patterns of focal gyrification and cognitive impairments in major psychiatric disorders.

### Distinct clinical manifestations

#### SCZ

Disturbed functional connectivity of the dorsal prefrontal cortex and anterior temporal or posterior inferior parietal cortices are reported as schizophrenia-specific impairments (especially in adulthood), possibly underlying their unique symptomatology^128^. As supported by the relationships of fronto-temporo-limbic disconnectivity with delusions, hallucinations, and disorganization symptoms in SCZ patients^117,118^, anomalous gyral patterns in the corresponding region may lead to the generation of positive psychotic symptoms. For verbal hallucinations in particular, aberrant connectivity of the arcuate fasciculus^136^ may lead to a series of abnormal activation, mainly in the language-related area as follows:^137^ (1) Hypofunction of the frontal midline structure [i.e., poor supplementary motor area activation^138^] activates the speech area; (2) increased activation in the primary auditory cortex^139^ converts the generated inner speech into external language stimuli; and (3) altered neural circuit of self-reflective processing [i.e., deviant activity in the right inferior parietal lobule^140^] gives otherness to the voice, resulting in the verbal hallucination. In addition, deviated patterns of frontoparietal gyrification may be involved in deficit syndrome in SCZ patients. The medial prefrontal and lateral parietal regions constitute the principal part of the DMN^141^, whose abnormalities can induce severe primary negative symptoms (e.g., anhedonia and avolition or apathy)^142,143^. However, a recent study demonstrates that functional dysconnectivity seen in SCZ patients seems to be driven by genetic diathesis rather than clinical expression^144^. Hence, these maps should be interpreted with caution.

#### BD

Relationships between the specific clinical symptoms of BD and gyrification pattern or brain connectivity have not been well documented.

#### ASD

Although ASD patients do not meet developmental milestones within typical limits^145^, they exhibit the frontotemporal hypo-gyrification pattern in early childhood. ASD patients have different developmental signs (e.g., pragmatic aspects of language use and lack of imagination) than SCZ patients^146^, which may be due to a distinct genetic mechanism. Autistic symptoms, such as impaired social reciprocity and communication, in ASD children may partly be explained by parietal gyral pattern changes. The findings of disturbed connectivity and deactivation in the somatosensory cortex^122,147^, which are correlated with autistic symptoms, support this postulation. Increased bottom-up and reduced top-down processing in the somatosensory cortex in ASD, presumed by increased long-range feedforward connectivity and reduced long-range feedback connectivity in the comparable area, can be implicated in some core features of ASD^148^.

#### MDD

Depressive symptoms in MDD patients were reported to correlate with gyral pattern changes in the lateral frontal cortices, lateral parietal cortices, and fusiform gyri^33,65^. Although depression and its-related pathopsychological characteristics (e.g., depressive rumination and over-general autobiographical memory) in MDD patients are associated with compromised connectivity in the affective network and DMN^149,150^, attenuated frontal-striatal reward network connectivity in proportion to depression severity is common in BD and MDD patients^151^. A recent cluster analysis demonstrated that a melancholia component is distributed equivalently across conventional psychiatric diagnoses^152^. Further studies are warranted to clarify whether depressive symptoms and their neural substrate are transdiagnostic or disease-specific.

## Future direction

### Reproducibility and generalizability of the findings

Although increasing neuroimaging evidence supports anomalous patterns of brain gyrification in several psychiatric disorders, there have been few longitudinal studies investigating the gyral pattern trajectory in each disorder or direct comparisons of gyrification patterns among major psychiatric disorders. Sample sizes in previous studies on cortical gyrification were small [hitherto largest sample of patients (*n* = 207)^26^] as opposed to those on other cortical indices [hitherto largest sample of patients (*n* > 4000)^153^ in a previous study investigating cortical thickness]. Thus, confirmatory studies using large-scale multi-site longitudinal brain MRI datasets are needed.

While we have adopted one-illness-one-pattern approach throughout this review, potential anatomical heterogeneity within each psychiatric disorder^154^ should also be tested in future gyrification studies using distinct subgroups.

Although the LGI is a widely accepted measure of brain gyrification, its linkage with other indicators remains unclear and the methodology has room for improvement. As the gyrification patterns may be closely interrelated with white matter integrity and implicate connectional characteristics of corresponding regions, confirming the relationship between LGI and advanced DTI measures [myelin content^155^ and corrected DTI indices^156^] may be useful. As detecting the disturbance of coordinated maturation among brain areas can provide more information about neurodevelopmental integrity than just exploring the localized changes in the brain^157^, gyrification-based connectomes adopting a graph theoretical approach are being investigated^158^. A novel indicator focusing on the gyral hinges^159^, considering a hierarchical organization within the gyral system, is also being developed.

Common and distinct biotypes on the basis of brain structural features among psychiatric disorders may provide the biological basis for making appropriate animal models. Together with recent progress in experimental biomedical research [e.g., Ferret models^73^] that will enable us to assess the models, we may be able to further our understanding of the pathophysiology of psychiatric disorders from the perspective of anomalous gyral patterns.

### Clinical significance

A previous study demonstrated that the deviated gyrification patterns can differentiate patients with schizophrenia from healthy controls with a favorable classification accuracy^160^. However, there have been only a few group comparisons of the gyrification pattern among psychiatric disorders [SCZ vs BD^64^; SCZ vs ASD^29^]. There are also only a few studies that examined the association between gyral pattern changes and later psychosis onset^17,158^ or antipsychotic treatment response in psychoses^161^. As mentioned above, establishing the relationships between deviated gyrification patterns and clinical phenotypes may aid in the development of biomarkers for both prediction (e.g., onset and treatment response) and diagnosis (e.g., diagnostic assistance and discrimination among psychiatric disorders). Such biomarkers will help optimize the prediction and diagnosis of psychiatric disorders.

Current diagnostic criteria for psychiatric disorders rely only on descriptive syndromes based upon a consensus of experts. In contrast, the Psychiatric Genomics Consortium previously reported cross-disorder effects of genetic variants, highlighting shared and distinct biological causes of major psychiatric disorders^6^. Similarly, as anomalous gyral patterns play a pleiotropic role in psychopathology, gyral findings may provide a novel nosology informed by neural circuits, which is beyond the present classifications in the psychiatric field.

## Conclusion

This review provides the transdiagnostic considerations of anomalous gyrification patterns in major psychiatric disorders. Diverse alterations of gyrification patterns at varying stages of major psychiatric disorders may reflect both molecular and biomechanical mechanisms. Patients with major psychiatric disorders exhibit commonly and differentially altered gyrification patterns, which suggests corresponding neural circuit changes involving the frontal and other brain regions, representing common (e.g., delayed infant milestones, social deficits, emotional dysregulation, and executive dysfunction) and distinct (e.g., autistic symptoms, psychotic symptoms, and deficit syndrome) clinical manifestations. A comprehensive understanding of the role of brain gyrification pattern in the pathophysiology of major psychiatric disorders may aid in the development of objective biomarkers for both prediction and diagnosis, as well as provide a novel nosology informed by neural circuits.

## Supplementary information

Supplementary Information
