# Supplementary material for: Anomalous brain gyrification patterns in major psychiatric disorders: a systematic review and transdiagnostic integration
Source: Transl Psychiatry. 2021 Mar 17;11:176. doi: 10.1038/s41398-021-01297-8 (PMC7969935; doi:10.1038/s41398-021-01297-8)
Supplement: Supplementary file 1 — Supplementary Information [file 41398_2021_1297_MOESM1_ESM.docx]

**Supplementary Information**

**Supplemental Table S1.** The Newcastle-Ottawa Scale

**Supplemental Table S2.** Summary of published MRI studies on individuals at high risk for psychosis

**Supplemental Table S3.** Summary of published MRI studies on SCZ patients

**Supplemental Table S4.** Summary of published MRI studies on BD patients

**Supplemental Table S5.** Summary of published MRI studies on MDD patients

**Supplemental Table S6.** Summary of published MRI studies on ASD patients

**Supplemental Table S7.** Summary of published MRI studies of the correlation between gyrification anomalies and clinical symptoms or cognitive performance

**Supplemental References**

**Supplemental Figure S1.** The gyrification findings in patients with major psychiatric disorders for each stage of illness compared with healthy controls

**Supplemental References**

**MRI studies counted in Supplemental Table S2**

Vogeley, K. et al. Right frontal hypergyria differentiation in affected and unaffected siblings from families multiply affected with schizophrenia: a morphometric mri study. *Am J Psychiatry.* **158**, 494-496 (2001).

Harris, J. M. et al. Abnormal cortical folding in high-risk individuals: a predictor of the development of schizophrenia? *Biol Psychiatry.* **56**, 182-189 (2004b).

Jou, R. J., Hardan, A. Y. & Keshavan, M. S. Reduced cortical folding in individuals at high risk for schizophrenia: a pilot study. *Schizophr Res.* **75**, 309-313 (2005).

Falkai, P. et al. Disturbed frontal gyrification within families affected with schizophrenia. *J Psychiatr Res.* **41**, 805-813 (2007).

Stanfield, A. C. et al. Increased right prefrontal cortical folding in adolescents at risk of schizophrenia for cognitive reasons. *Biol Psychiatry*. **63**, 80-85 (2008).

Dauvermann, M. R. et al. Relationship between gyrification and functional connectivity of the prefrontal cortex in subjects at high genetic risk of schizophrenia. *Curr Pharm Des.* **18**, 434-442 (2012).

Tepest, R. et al. Morphometry of structural disconnectivity indicators in subjects at risk and in age-matched patients with schizophrenia. *Eur Arch Psychiatry Clin Neurosci*. **263**, 15-24 (2013).

Nanda, P. et al. Local gyrification index in probands with psychotic disorders and their first-degree relatives. *Biol Psychiatry*. **76**, 447-455 (2014).

Bakker, G. et al. Cortical Morphology Differences in Subjects at Increased Vulnerability for Developing a Psychotic Disorder: A Comparison between Subjects with Ultra-High Risk and 22q11.2 Deletion Syndrome. *PLoS One.* **11**, e0159928 (2016).

Sasabayashi, D. et al. Increased Occipital Gyrification and Development of Psychotic Disorders in Individuals With an At-Risk Mental State: A Multicenter Study. *Biol Psychiatry*. **82**, 737-745 (2017b).

Das, T. et al. Disorganized Gyrification Network Properties During the Transition to Psychosis. *JAMA Psychiatry*. **75**, 613-622 (2018).

Damme, K. S. F. et al. Cortical Morphometry in the Psychosis Risk Period: A Comprehensive Perspective of Surface Features. *Biol Psychiatry Cogn Neurosci Neuroimaging*. **4**, 434-443 (2019).

Sasabayashi, D. et al. Increased brain gyrification in the schizophrenia spectrum. *Psychiatry Clin Neurosci*. **74**, 70-76 (2020).

**MRI studies counted in Supplemental Table S3**

Kulynych, J. J., Luevano, L. F., Jones, D.W. & Weinberger, D. R. Cortical abnormality in schizophrenia: an in vivo application of the gyrification index. *Biol Psychiatry*. **41**, 995-999 (1997).

Highley, J. R. et al. Sex-dependent effects of schizophrenia: an MRI study of gyral folding, and cortical and white matter volume. *Psychiatry Res*. **124**, 11-23 (2003).

Sallet, P. C. et al. Reduced cortical folding in schizophrenia: an MRI morphometric study. *Am J Psychiatry*. **160**, 1606-1613 (2003).

White, T., Andreasen, N. C., Nopoulos, P. & Magnotta, V. Gyrification abnormalities in childhood- and adolescent-onset schizophrenia. *Biol Psychiatry*. **54**, 418-426 (2003).

Harris, J. M. et al. Gyrification in first-episode schizophrenia: a morphometric study. *Biol Psychiatry*. **55**, 141-147 (2004a).

Narr, K. L. et al. Abnormal gyral complexity in first-episode schizophrenia. *Biol Psychiatry*. **55**, 859-867 (2004).

Wiegand, L. C. et al. An in vivo MRI study of prefrontal cortical complexity in first-episode psychosis. *Am J Psychiatry*. **162**, 65-70 (2005).

Bonnici, H. M. et al. Pre-frontal lobe gyrification index in schizophrenia, mental retardation and comorbid groups: an automated study. *Neuroimage*. **35**, 648-654 (2007).

Falkai, P. et al. Disturbed frontal gyrification within families affected with schizophrenia. *J Psychiatr Res*. **41**, 805-813 (2007).

Cachia, A. et al. Cortical folding abnormalities in schizophrenia patients with resistant auditory hallucinations. *Neuroimage*. **39**, 927-935 (2008).

Penttilä, J. et al. Global and temporal cortical folding in patients with early-onset schizophrenia. *J Am Acad Child Adolesc Psychiatry*. **47**, 1125-1132 (2008).

Janssen, J. et al. Gyral and sulcal cortical thinning in adolescents with first episode early-onset psychosis. *Biol Psychiatry*. **66**, 1047-1054 (2009).

McIntosh, A. M. et al. Prefrontal gyral folding and its cognitive correlates in bipolar disorder and schizophrenia. *Acta Psychiatr Scand*. **119**, 192-198 (2009).

Schultz, C. C. et al. Increased parahippocampal and lingual gyrification in first-episode schizophrenia. *Schizophr Res*. **123**, 137-144 (2010).

Haukvik, U.K. et al. Cortical folding in Broca's area relates to obstetric complications in schizophrenia patients and healthy controls. *Psychol Med*. **42**, 1329-1337 (2012).

Palaniyappan, L. & Liddle, P. F. Aberrant cortical gyrification in schizophrenia: a surface-based morphometry study. *J Psychiatry Neurosci*. **37**, 399-406 (2012).

Ronan, L. et al. Consistency and interpretation of changes in millimeter-scale cortical intrinsic curvature across three independent datasets in schizophrenia. *Neuroimage*. **63**, 611-621 (2012).

Bartholomeusz, C. F. et al. Sulcogyral patterns and morphological abnormalities of the orbitofrontal cortex in psychosis. *Prog Neuropsychopharmacol Biol Psychiatry*. **44**, 168-177 (2013).

Palaniyappan, L. et al. Gyrification of Broca's region is anomalously lateralized at onset of schizophrenia in adolescence and regresses at 2 year follow-up. *Schizophr Res*. **147**, 39-45 (2013).

Schultz, C. C. et al. The visual cortex in schizophrenia: alterations of gyrification rather than cortical thickness--a combined cortical shape analysis. *Brain Struct Funct*. **218**, 51-58 (2013).

Tepest, R. et al. Morphometry of structural disconnectivity indicators in subjects at risk and in age-matched patients with schizophrenia. *Eur Arch Psychiatry Clin Neurosci*. **263**, 15-24 (2013).

Janssen, J. et al. Cortical morphology of adolescents with bipolar disorder and with schizophrenia. *Schizophr Res*. **158**, 91-99 (2014).

Nanda, P. et al. Local gyrification index in probands with psychotic disorders and their first-degree relatives. *Biol Psychiatry*. **76**, 447-455 (2014).

Nesvag, R. et al. Reduced brain cortical folding in schizophrenia revealed in two independent samples. *Schizophr Res*. **152**, 333-338 (2014).

Palaniyappan, L. & Liddle, P. F. Diagnostic discontinuity in psychosis: a combined study of cortical gyrification and functional connectivity. *Schizophr Bull*. **40**, 675-684 (2014).

Nenadic, I. et al. Prefrontal gyrification in psychotic bipolar I disorder vs. schizophrenia. *J Affect Disord*. **185**, 104-107 (2015).

Hirjak, D. et al. Neuroanatomical Markers of Neurological Soft Signs in Recent-Onset Schizophrenia and Asperger-Syndrome. *Brain Topogr*. **29**, 382-394 (2016).

Cao, B. et al. Lifespan Gyrification Trajectories of Human Brain in Healthy Individuals and Patients with Major Psychiatric Disorders. *Sci Rep*. **7**, 511 (2017).

Sasabayashi, D. et al. Increased Frontal Gyrification Negatively Correlates with Executive Function in Patients with First-Episode Schizophrenia. *Cereb Cortex*. **27**, 2686-2694 (2017a).

Kubera, K. M. et al. Cortical folding abnormalities in patients with schizophrenia who have persistent auditory verbal hallucinations. *Eur Neuropsychopharmacol*. **28**, 297-306 (2018).

Nelson, E. A., White, D. M., Kraguljac, N. V. & Lahti, A. C. Gyrification Connectomes in Unmedicated Patients With Schizophrenia and Following a Short Course of Antipsychotic Drug Treatment. *Front Psychiatry.* **9**, 699 (2018).

Spalthoff, R., Gaser, C. & Nenadic, I. Altered gyrification in schizophrenia and its relation to other morphometric markers. *Schizophr Res*. **202**, 195-202 (2018).

Zuliani, R. et al. Increased gyrification in schizophrenia and non affective first episode of psychosis. *Schizophr Res*. **193**, 269-275 (2018).

Takayanagi, Y. et al. Altered brain gyrification in deficit and non-deficit schizophrenia. *Psychol Med*. **49**, 573-580 (2019).

**MRI studies counted in Supplemental Table S4**

Liao, Y. L. et al. Cortical complexity analysis of patients with bipolar disorder using three-dimensional gyrification index. *Conf Proc IEEE Eng Med Biol Soc*. 3933-3936 (2008).

McIntosh, A. M. et al. Prefrontal gyral folding and its cognitive correlates in bipolar disorder and schizophrenia. *Acta Psychiatr Scand*. **119**, 192-198 (2009).

Mirakhur, A. et al. Changes in gyrification over 4 years in bipolar disorder and their association with the brain-derived neurotrophic factor valine(66) methionine variant. *Biol Psychiatry.* **66**, 293-297 (2009).

Penttilä, J. et al. Cortical folding in patients with bipolar disorder or unipolar depression. *J Psychiatry Neurosci.* **34**, 127-135 (2009a).

Penttilä, J. et al. Cortical folding difference between patients with early-onset and patients with intermediate-onset bipolar disorder. *Bipolar Disord*. **11**, 361-370 (2009b).

Janssen, J. et al. Cortical morphology of adolescents with bipolar disorder and with schizophrenia. *Schizophr Res*. **158**, 91-99 (2014).

Nanda, P. et al. Local gyrification index in probands with psychotic disorders and their first-degree relatives. *Biol Psychiatry*. **76**, 447-455 (2014).

Palaniyappan, L. & Liddle, P. F. Diagnostic discontinuity in psychosis: a combined study of cortical gyrification and functional connectivity. *Schizophr Bull*. **40**, 675-684 (2014).

Nenadic, I. et al. Prefrontal gyrification in psychotic bipolar I disorder vs. schizophrenia. *J Affect Disord*. **185**, 104-107 (2015).

Cao, B. et al. Lifespan Gyrification Trajectories of Human Brain in Healthy Individuals and Patients with Major Psychiatric Disorders. *Sci Rep*. **7**, 511 (2017).

Sarrazin, S. et al. Neurodevelopmental subtypes of bipolar disorder are related to cortical folding patterns: An international multicenter study. *Bipolar Disord*. **20**, 721-732 (2018).

**MRI studies counted in Supplemental Table S5**

Penttilä, J. et al. Cortical folding in patients with bipolar disorder or unipolar depression. *J Psychiatry Neurosci.* **34**, 127-135 (2009a).

Zhang, Y. et al. Decreased gyrification in major depressive disorder. *Neuroreport*. **20**, 378-380 (2009).

Nixon, N. L. et al. Biological vulnerability to depression: linked structural and functional brain network findings. *Br J Psychiatry.* **204**, 283-289 (2014).

Peng, D. et al. Surface vulnerability of cerebral cortex to major depressive disorder. *PLoS One.* **10**, e0120704 (2015).

Cao, B. et al. Lifespan Gyrification Trajectories of Human Brain in Healthy Individuals and Patients with Major Psychiatric Disorders. *Sci Rep*. **7**, 511 (2017).

Han, K. M. et al. Local gyrification index in patients with major depressive disorder and its association with tryptophan hydroxylase-2 (TPH2) polymorphism. *Hum Brain Mapp*. **38**, 1299-1310 (2017).

Depping, M. S. et al. Common and distinct patterns of abnormal cortical gyrification in major depression and borderline personality disorder. *Eur Neuropsychopharmacol*. **28**, 1115-1125 (2018).

Schmitgen, M. M. et al. Aberrant cortical neurodevelopment in major depressive disorder. *J Affect Disord*. **243**, 340-347 (2019).

**MRI studies counted in Supplemental Table S6**

Hardan, A. Y., Jou, R. J., Keshavan, M. S., Varma, R. & Minshew, N. J. Increased frontal cortical folding in autism: a preliminary MRI study. *Psychiatry Res*. **131**, 263-268 (2004).

Kates, W. R., Ikuta, I. & Burnette, C. P. Gyrification patterns in monozygotic twin pairs varying in discordance for autism. *Autism Res*. **2**, 267-278 (2009).

Jou, R. J., Minshew, N. J., Keshavan, M. S. & Hardan, A. Y. Cortical gyrification in autistic and Asperger disorders: a preliminary magnetic resonance imaging study. *J Child Neurol*. **25**, 1462-1467 (2010).

Schaer, M. et al. Decreased frontal gyrification correlates with altered connectivity in children with autism. *Front Hum Neurosci.* **7**, 750 (2013).

Wallace, G. L. et al. Increased gyrification, but comparable surface area in adolescents with autism spectrum disorders. *Brain*. **136**, 1956-1967 (2013).

Libero, L. E., DeRamus, T. P., Deshpande, H. D. & Kana, R. K. Surface-based morphometry of the cortical architecture of autism spectrum disorders: volume, thickness, area, and gyrification. *Neuropsychologia*. **62**, 1-10 (2014).

Blanken, L. M. et al. Cortical morphology in 6- to 10-year old children with autistic traits: a population-based neuroimaging study. *Am J Psychiatry*. **172**, 479-486 (2015).

Schaer, M., Kochalka, J., Padmanabhan, A., Supekar, K. & Menon, V. Sex differences in cortical volume and gyrification in autism. *Mol Autism.* **6**, 42 (2015).

Ecker, C. et al. Relationship Between Cortical Gyrification, White Matter Connectivity, and Autism Spectrum Disorder. *Cereb Cortex*. **26**, 3297-3309 (2016).

Hirjak, D. et al. Neuroanatomical Markers of Neurological Soft Signs in Recent-Onset Schizophrenia and Asperger-Syndrome. *Brain Topogr*. **29**, 382-394 (2016).

Koolschijn, P. & Geurts, H. M. Gray Matter Characteristics in Mid and Old Aged Adults with ASD. *J Autism Dev Disord*. **46**, 2666-2678 (2016).

Yang, D. Y., Beam, D., Pelphrey, K. A., Abdullahi, S. & Jou, R. J. Cortical morphological markers in children with autism: a structural magnetic resonance imaging study of thickness, area, volume, and gyrification. *Mol Autism.* **7**, 11 (2016).

Duret, P. et al. Gyrification changes are related to cognitive strengths in autism. *Neuroimage Clin.* **20**, 415-423 (2018).

Maier, S. et al. Cortical properties of adults with autism spectrum disorder and an IQ>100. *Psychiatry Res Neuroimaging*. **279**, 8-13 (2018).

Pappaianni, E. et al. Three shades of grey: detecting brain abnormalities in children with autism using source-, voxel- and surface-based morphometry. *Eur J Neurosci*. **47**, 690-700. (2018)

Kohli, J. S. et al. Local Cortical Gyrification is Increased in Children With Autism Spectrum Disorders, but Decreases Rapidly in Adolescents. *Cereb Cortex*. **29**, 2412-2423 (2019).

Libero, L. E., Schaer, M., Li, D. D., Amaral, D. G. & Nordahl, C. W. A Longitudinal Study of Local Gyrification Index in Young Boys With Autism Spectrum Disorder. *Cereb Cortex*. **29**, 2575-2587 (2019).

**MRI studies counted in Supplemental Table S7**

Sallet, P. C. et al. Reduced cortical folding in schizophrenia: an MRI morphometric study. *Am J Psychiatry*. **160**, 1606-1613 (2003).

Stanfield, A. C. et al. Increased right prefrontal cortical folding in adolescents at risk of schizophrenia for cognitive reasons. *Biol Psychiatry*. **63**, 80-85 (2008).

McIntosh, A. M. et al. Prefrontal gyral folding and its cognitive correlates in bipolar disorder and schizophrenia. *Acta Psychiatr Scand*. **119**, 192-198 (2009).

Kates, W. R., Ikuta, I. & Burnette, C. P. Gyrification patterns in monozygotic twin pairs varying in discordance for autism. *Autism Res*. **2**, 267-278 (2009).

Schaer, M. et al. Decreased frontal gyrification correlates with altered connectivity in children with autism. *Front Hum Neurosci.* **7**, 750 (2013).

Hirjak, D. et al. Neuroanatomical Markers of Neurological Soft Signs in Recent-Onset Schizophrenia and Asperger-Syndrome. *Brain Topogr*. **29**, 382-394 (2016).

Yang, D. Y., Beam, D., Pelphrey, K. A., Abdullahi, S. & Jou, R. J. Cortical morphological markers in children with autism: a structural magnetic resonance imaging study of thickness, area, volume, and gyrification. *Mol Autism.* **7**, 11 (2016).

Sasabayashi, D. et al. Increased Frontal Gyrification Negatively Correlates with Executive Function in Patients with First-Episode Schizophrenia. *Cereb Cortex*. **27**, 2686-2694 (2017a).

Depping, M. S. et al. Common and distinct patterns of abnormal cortical gyrification in major depression and borderline personality disorder. *Eur Neuropsychopharmacol*. **28**, 1115-1125 (2018).

Kubera, K. M. et al. Cortical folding abnormalities in patients with schizophrenia who have persistent auditory verbal hallucinations. *Eur Neuropsychopharmacol*. **28**, 297-306 (2018).

Kohli, J. S. et al. Local Cortical Gyrification is Increased in Children With Autism Spectrum Disorders, but Decreases Rapidly in Adolescents. *Cereb Cortex*. **29**, 2412-2423 (2019).

Libero, L. E., Schaer, M., Li, D. D., Amaral, D. G. & Nordahl, C. W. A Longitudinal Study of Local Gyrification Index in Young Boys With Autism Spectrum Disorder. *Cereb Cortex*. **29**, 2575-2587 (2019).

Schmitgen, M. M. et al. Aberrant cortical neurodevelopment in major depressive disorder. *J Affect Disord*. **243**, 340-347 (2019).

Takayanagi, Y. et al. Altered brain gyrification in deficit and non-deficit schizophrenia. *Psychol Med*. **49**, 573-580 (2019).

**Supplemental Figure S1.** The gyrification findings in patients with major psychiatric disorders for each stage of illness compared with healthy controls

Black arrow: Dominant direction of gyral pattern changes based on previous gyral findings being weighted according to the number of studies, sample size for studies, representativeness of cases, and methodological advantage to assess gyral patterns

Abbreviations: BD, bipolar disorder; MDD, major depressive disorder; SCZ, schizophrenia
